# Oral manifestations of COVID-19 in unvaccinated patients: a cross-sectional study

**DOI:** 10.1186/s12903-023-03325-z

**Published:** 2023-09-27

**Authors:** Tatiana Jorge Fernandes, Maria Ogrzewalska, Ezequias Batista Martins, Marilda Agudo Mendonça Teixeira de Siqueira, Patrícia Brasil, Guilherme Amaral Calvet

**Affiliations:** 1 Evandro Chagas National Institute of Infectious Diseases, Oswaldo Cruz Foundation, Rio de Janeiro, Brazil; 2 Oswaldo Cruz Institute, Oswaldo Cruz Foundation, Rio de Janeiro, Rio de Janeiro, Brazil; 3 SARS-CoV National Reference Laboratory for the Brazilian Ministry of Health (MoH) and Regional Reference Laboratory in Americas for the Pan-American Health Organization, Washington, USA; 4 Acute Febrile Illnesses Laboratory, Evandro Chagas National Institute of Infectious Diseases - Oswaldo Cruz Foundation, Av. Brasil, 4365, Rio de Janeiro, 21045-900 Manguinhos Brazil

**Keywords:** SARS-CoV-2, COVID-19, Saliva, Oral manifestations, Oral cavity, Xerostomia

## Abstract

**Background:**

Early studies have highlighted the possible development of dysgeusia and anosmia in severe acute respiratory syndrome coronavirus 2 (SARS-CoV-2) infection, and these manifestations should be considered a potential indication of coronavirus disease 19 (COVID-19). As potential contributors to these symptoms, dentists should perform careful oral and oropharyngeal examinations and document suspicious oral lesions in patients with COVID-19, especially in those who complain of loss of taste and smell. The study’s objective was to assess the prevalence of oral manifestations among ambulatory unvaccinated symptomatic patients with suspected COVID-19 during the acute phase of the disease.

**Methods:**

This cross-sectional study evaluated oral manifestations in adults (aged ≥ 18 years) with suspected and confirmed SARS-CoV-2 infection. Chi-square and Fisher’s exact tests were used to compare data between the groups (rRT-PCR-positive and rRT-PCR-negative patients).

**Results:**

One hundred thirty-six participants were included. Most were female (n = 79; 58.1%), with a mean age of 39.53 (± 14.17) years. Of these, 54 (39.7%) had a positive rRT-PCR test, and 82 (60.3%) had negative rRT-PCR results. Oral manifestations were observed in 40 participants (74.1%) in the rRT-PCR-positive group and 67 participants (81.7%) in the rRT-PCR-negative group. The most common oral manifestations were xerostomia (n = 85; 62.5%) and dysgeusia/ageusia (n = 57; 41.9%). Different rates of gingivitis (n = 12; 22.2% vs. n = 5; 6.1%; p = 0.005) and halitosis (n = 7; 13.0% vs. n = 1; 1.2%; p = 0.007) were observed between the rRT-PCR-positive and -negative groups, respectively. Mouth ulcers, glossitis, tongue coating, and petechiae were reported in both groups without significant differences.

**Conclusions:**

A high prevalence of oral manifestations was observed in symptomatic patients with suspected or confirmed COVID-19.

**Clinical Relevance:**

This study highlights the importance of routine oral examinations by dentists as part of the multidisciplinary care of COVID-19 patients.

## Introduction

Severe acute respiratory syndrome coronavirus 2 (SARS-CoV-2) is a highly contagious zoonotic virus that originated in Wuhan, China, in December 2019 [1]. Coronaviruses are a family of viruses that cause respiratory infections, including the new coronavirus (SARS-CoV-2). The resulting coronavirus disease 19 (COVID-19) was declared a global health emergency on January 30, 2020, and a pandemic on March 11, 2020, by the World Health Organization (WHO) [2, 3]. As a result, there is a growing concern in multiple specialties, including dentistry, due to its high virulence and transmission routes through salivary aerosols. Research addressing crucial unknowns regarding the clinical severity, extent of transmission, and treatment options has been ongoing worldwide since the inception of the disease. The primary response strategy includes limiting human-to-human transmission [4].

As the oral cavity is one of the first interfaces between the environment and the body, there is a high potential that this route of colonization and viral infection is critical for the emergence of COVID-19 [5]. Approximately 99% of saliva is water; the rest comprises components responsible for digestion, taste, buffering, remineralization balance, and antimicrobial activity. The remaining components include a mixture of desquamated oral epithelial cells, salivary secretions from major and minor glands, crevicular fluid, and microorganisms, including bacteria, fungi, and viruses. The oral cavity is an entrance and outlet of the body, and saliva plays a role in the early diagnosis and close contact transmission of infectious diseases [6].

Accurate diagnosis is essential to control the outbreak and to propose measures related to the prevention and prognosis of infection. Saliva is a reliable, noninvasive specimen for diagnosing patients with suspected COVID-19 [7]. Furthermore, oral fluid can be self-collected by subjects and can be easily managed, dramatically reducing the risk of viral transmission for healthcare workers [8].

Early studies have highlighted the possible development of dysgeusia and anosmia in SARS-CoV-2 infection [9, 10], and these manifestations should be considered a potential indication of COVID-19. As potential contributors to these symptoms, dentists should perform careful oral and oropharyngeal examinations and document suspicious oral lesions in patients with COVID-19, especially in those who complain of loss of taste and smell.

A cross-sectional study conducted in Spain with 666 hospitalized COVID-19 patients reported oral findings in 78 cases (25.7%), including transient lingual papillitis (11.5%), glossitis with lateral indentations (6.6%), aphthous stomatitis (6.9%), glossitis with patchy depapillation (3.9%), and mucositis (3.9%) [11].

A review of oral manifestations in COVID-19 patients reported the presence of several oral lesions, including ulcers, erosions, bullae, vesicles, pustules, fissured or depapillated tongue, macules, papules, plaques, pigmentation, halitosis, whitish areas, hemorrhagic crust, necrosis, petechiae, swelling, erythema, and spontaneous bleeding [12]. In addition, a recent systematic review found a higher prevalence of oral manifestations of SARS-CoV-2, especially xerostomia, ulcerations, and taste alterations, in patients with active disease [13].

This study aimed to assess the prevalence of oral manifestations among ambulatory unvaccinated symptomatic patients with suspected COVID-19 during the acute phase of the disease.

## Materials and methods

This observational cross-sectional study was conducted at the outpatient service for acute febrile illnesses at the Evandro Chagas National Institute of Infectious Diseases, Fiocruz, Rio de Janeiro, Brazil. Males and females (pregnant or nonpregnant) aged ≥ 18 years with signs and symptoms of SARS-CoV-2 infection or in contact with people with confirmed COVID-19 were screened and enrolled in the study after signing informed consent. The study excluded individuals who refused nasopharynx/oropharynx and salivary sample collection.

The patients were interviewed by a dentist using an electronic case report form (eCRF) based on the Research Electronic Data Capture (REDCap) platform [14, 15] hosted at the Evandro Chagas National Institute of Infectious Diseases. An enrollment questionnaire collected detailed baseline sociodemographic features, comorbidities, clinical signs, and symptoms. A thorough oral examination was performed using a mouth mirror by the same dentist wearing complete personal protective equipment. The gingival condition was assessed through a comprehensive dental examination, encompassing a thorough assessment of the gums and surrounding tissues. Specifically, the dentist conducted a visual examination to inspect the gums and surrounding tissues for signs of inflammation, redness, swelling, or discoloration.

Additionally, she meticulously searched for any abnormalities, such as sores or growths. Before the oral examination, the patients used a mouthwash containing chlorhexidine (Periogard© from Colgate) for 15 s. Data were recorded on an oral clinical examination form.

### Laboratory analysis and confirmation

Nasopharynx/oropharynx swabs and saliva samples were tested using rRT-PCR for SARS-CoV-2 detection. Nurses collected nasopharyngeal and oropharyngeal swabs. The same dentist collected saliva by asking the participants to accumulate saliva (at least 1–2 mL) in the mouth for one minute and then spit it into a sterile container without coughing or clearing their throats. Briefly, samples were screened for SARS-CoV-2 by rRT-PCR to amplify the E gene and RdRp region of the Orf1ab gene of SARS-CoV-2 using the SARS-CoV-2 E/RP molecular kit (Bio-Manguinhos, Rio de Janeiro, Brazil). A cycle threshold (Ct) less than 40 was classified as positive. Participants with a detectable rRT-PCR test in nasopharyngeal/oropharyngeal swabs and/or saliva were assigned to the positive rRT-PCR group. Conversely, the negative rRT-PCR group included participants with undetectable rRT-PCR results in nasopharyngeal/oropharyngeal swabs and saliva specimens.

### Statistical analyses

The sample size calculation was performed in the epiDisplay package of R 4.1.1 software to compare the proportions between the two groups, with a significance level of 5% and power of 90% and at least 55 people in each group to state the difference between these proportions, which reached a sample size of 110 participants.

Sociodemographic and clinical variables were determined using frequencies and proportions for categorical variables and means with standard deviations for continuous variables. The chi-square test or Fisher’s exact test for categorical variables was conducted to assess differences in oral manifestations between the rRT-PCR-positive and rRT-PCR-negative groups, as appropriate. Additionally, the odds ratio (OR) and confidence interval were calculated using a logistic regression model. Statistical analyses were performed using IBM SPSS Statistics 22.0. The recommendations of the Strengthening the Reporting of Observational Studies in Epidemiology (STROBE) guidelines were applied for reporting.

## Results

**Fig. 1:**
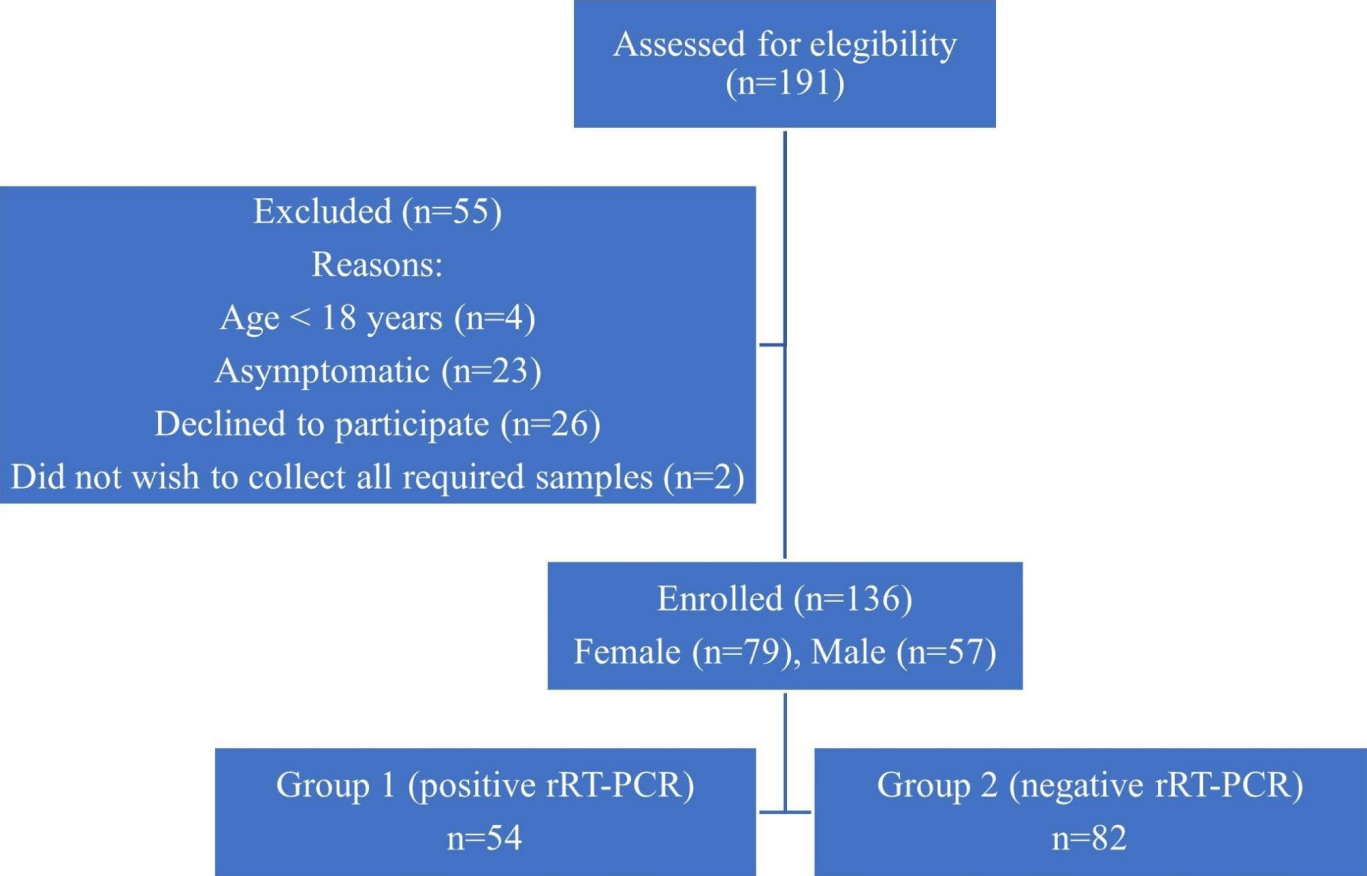
Flow diagram of the study

Between October 2020 and September 2021, 191 individuals were assessed for eligibility, and 136 participants were enrolled in the study (Fig. 1). Of these, 54 (39.7%) had a positive rRT-PCR test result, and 82 (60.3%) had negative rRT-PCR results. Samples were collected at a mean of 4.56 (± 2.90) days after the onset of symptoms.

### Sociodemographic and clinical characteristics

Table 1 summarizes all participants’ sociodemographic and clinical characteristic profiles according to the rRT-PCR results. Most of the participants were female (n = 79; 58.1%). The mean age of all study participants was 39.53 (± 14.17) years; most were white (n = 60; 44.1%) and had a university/postgraduation level of education (n = 70; 51.5%). Several comorbidities were reported in both groups.

**Table 1:** Baseline characteristics of 136 study participants

Characteristics	Positive rRT-PCR N (%)	Negative rRT-PCR N (%)
	(n = 54)	(n = 82)
**Mean age (± SD)**	41.87 (± 14.04)	37.99 (± 14.13)
**Gender**		
Male	27 (50)	30 (36.6)
Female	27 (50)	52 (63.4)
**Race/ethnicity, n (%)**		
White	25 (46.3)	35 (42.7)
Black	8 (14.8)	17 (20.7)
Mixed	21 (38.9)	25 (30.5)
Indigenous	-	1 (1.2)
Yellow	-	4 (4.9)
**Highest formal educational attendance, n (%)**		
University/post-graduation	24 (44.4)	46 (56.1)
High school/technical college	24 (44.4)	21 (25.6)
Lower Secondary	3 (5.6)	2 (2.4)
Primary	1 (1.9)	2 (2.4)
Less than primary	2 (3.7)	10 (12.2)
Missing	-	1 (1.2)
**Self-reported history of preexisting diseases, n (%)**		
Obesity	7 (12.9)	11 (13.4)
Cancer	3 (5.6)	2 (2.4)
Diabetes mellitus	3 (5.6)	5 (6.1)
HIV infection	8 (14.8)	11 (13.4)
Cardiovascular disease	5 (9.2)	6 (7.3)
Asthma requiring medication	2 (3.7)	7 (8.5)
Lung disease (other than asthma)	4 (7.4)	6 (7.3)
Chronic liver disease	2 (3.7)	-
Chronic hematologic disease	2 (3.7)	3 (3.6)
Chronic kidney disease	3 (5.6)	4 (4.9)
Chronic neurological disease	2 (3.7)	8 (9.8)
Received transplant	1 (1.8)	2 (2.4)
Hypertension	13 (24.1)	12 (14.6)
Chronic rhinitis	10 (18.5)	14 (17.1)
Thyroid disease	8 (14.8)	6 (7.3)
**Current smoker**	6 (11.1)	10 (12.2)

SD: standard deviation; N: number; %: percent; rRT-PCR: real-time reverse-transcriptase polymerase chain reaction

### Oral signs and symptoms

Oral manifestations at enrollment included xerostomia (dry mouth), taste alterations (dysgeusia/ageusia), gingivitis, glossitis, halitosis, mouth ulcers, petechiae, and tongue coating (Table 2).

**Table 2:** Oral manifestations of 136 study participants

Oral Manifestations	Symptomatic positive rRT-PCR N (%)	Symptomatic negative rRT-PCR N (%)	p value	Crude OR	95% CI
Any oral manifestations					
No	14 (25.9)	15 (18.3)		1.00	
Yes	40 (74.1)	67 (81.7)	0.288	0.64	0.28–1.46
**Xerostomia**					
No	25 (46.3)	26 (31.7)		1.00	
Yes	29 (53.7)	56 (68.3)	0.086	0.54	0.27–1.09
**Dysgeusia/ageusia**					
No	30 (55.6)	49 (59.8)		1.00	
Yes	24 (44.4)	33 (40.2)	0.627	1.19	0.59–2.38
**Gingivitis**					
No	42 (77.8)	77 (93.9)		1.00	
Yes	12 (22.2)	5 (6.1)	**0.005**	**4.40**	**1.45–13.34**
**Halitosis**					
No	47 (87.0)	81 (98.8)		1.00	
Yes	7 (13.0)	1 (1.2)	0.007*	**12.06**	**1.44–101.10**
**Mouth ulcers (self-reported)**					
No	49 (90.7)	70 (85.4)		1.00	
Yes	5 (9.3)	12 (14.6)	0.354	0.60	0.20–1.80
**Mouth ulcers (clinical exam)**					
No	54 (100.0)	79 (96.3)		-	
Yes	0 (0.0)	3 (3.7)	-	-	-
**Glossitis**					
No	49 (90.7)	74 (90.2)		1.00	
Yes	5 (9.3)	8 (9.8)	0.923	0.94	0.29–3.05
**Tongue coating**					
No	50 (92.6)	80 (97.6)		1.00	
Yes	4 (7.4)	2 (2.4)	0.214*	3.20	0.57–18.12
**Petechiae**					
No	50 (92.6)	79 (96.3)		1.00	
Yes	4 (7.4)	3 (3.7)	0.435*	2.11	0.45–9.81

rRT-PCR: real-time reverse-transcriptase polymerase chain reaction; N: number; %: percent; The bold values represent the statistically significant results; The chi-square test was used unless otherwise noted; * Fisher’s exact test was used. OR: Odds Ratio; CI: Confidence interval

Xerostomia (53.7%) was the most frequent oral manifestation in the symptomatic positive rRT-PCR group, followed by dysgeusia/ageusia (44.4%), gingivitis (22.2%), halitosis (13.0%), self-reported mouth ulcers (10%), and glossitis (9.3%). In the symptomatic negative rRT-PCR group, xerostomia (68.3%) was the primary oral manifestation, followed by dysgeusia/ageusia (40.2%), self-reported mouth ulcers (14.6%), glossitis (9.8%), and gingivitis (6.1%). Mouth ulcers were self-reported in 12 participants (14.6%) but were observed in only three participants (3.7%) in the negative group when the oral examination was performed. Participants with detectable SARS-CoV-2 rRT-PCR were 4.4 times more likely to have gingivitis (OR = 4.4, 95% CI: 1.45 to 13.34) and 12.06 times more likely to have halitosis (OR = 12.06, 95% CI: 1.44 to 101.10) than their counterparts with no detectable SARS-CoV-2 rRT-PCR (Table 2).

**Fig. 2:**
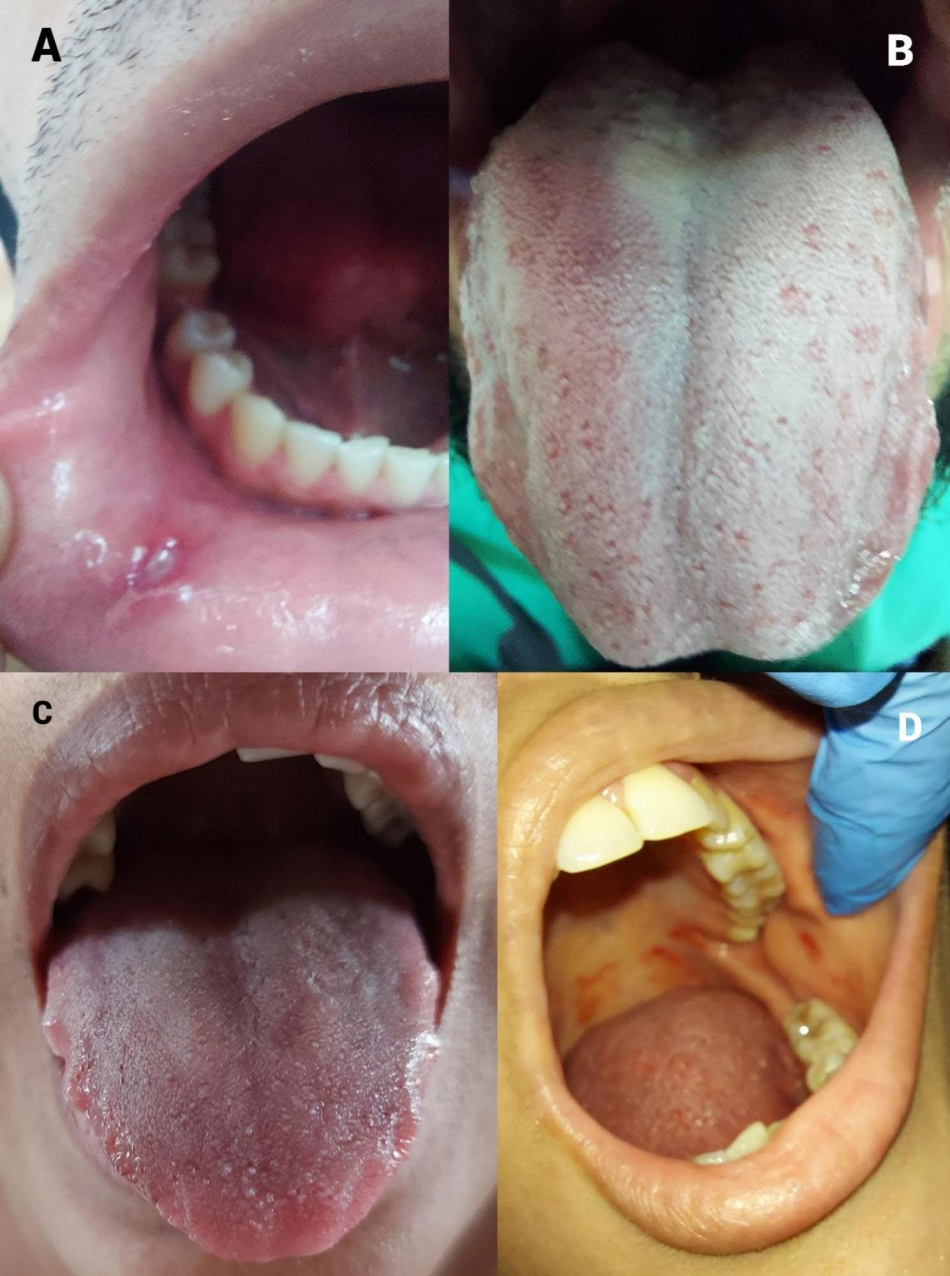
Clinical presentations and diagnoses. (**A**) A male participant presented with a painful lesion on the inside of the bottom lip, exhibiting a clinical appearance of a white bump surrounded by a red, inflamed border. The center of the lesion appeared grayish-white. Clinical diagnosis: Mouth ulcer. (**B**) A male participant had a tongue surface coated with a thick whitish film and a fuzzy texture. Clinical diagnosis: Tongue coating. (**C**) A female participant presented a tongue with deep grooves on the surface running perpendicular to its length. Clinical diagnosis: Glossitis with indentations. (**D**) A female participant exhibited small pinpoint reddish-purple spots on the soft palate and buccal mucosa. Clinical diagnosis: Oral petechial lesions. **Note**: The clinical descriptions and diagnoses mentioned above were based on careful examination and evaluation by a qualified dentist

Figure 2 illustrates the oral manifestations along with their clinical presentations and diagnoses. Mouth ulcer (Fig. 2A), tongue coating (Fig. 2B), glossitis with indentations (teeth marks on the sides) (Fig. 2C), and oral petechial lesions (Fig. 2D). All these oral lesions were observed in both groups.

## Discussion

The term “oral manifestations” has not yet been conclusively associated with pathognomonic features of COVID-19. Sarasati et al. [16]. found inconsistencies and the absence of pathognomonic features in previous studies on oral lesions in COVID-19 patients. Soares et al. [17]. suggest a potential causality due to angiotensin-converting enzyme 2 (ACE-2) in oral epithelial tissue. ACE-2 is suspected to serve as the initial receptor for developing oral lesions in SARS-CoV-2-infected patients. With ACE2 expression, oral tissues could facilitate direct SARS-CoV-2 invasion, contributing to its pathogenesis and potentially enhancing human-to-human transmission rates [6, 16]. Limongelli et al. [18]. present evidence that SARS-CoV-2 may persist in the oral epithelium/mucosa beyond the acute phase, potentially leading to lesions. However, the underlying pathogenetic mechanism requires further clarification. This disparity raises questions about whether these oral lesions directly result from SARS-CoV-2 (with identifiable specific cause and effect or pathognomonic features) or are merely coincidental.

In this study, xerostomia and dysgeusia/ageusia were the most frequent oral manifestations in symptomatic participants regardless of the molecular confirmation of COVID-19. These results were similar to those of another study conducted with 128 nonhospitalized patients with COVID-19 confirmed by rRT-PCR, where 56% of patients reported xerostomia as the most frequent oral manifestation, followed by gustatory dysfunction (32.8%) [19].

Gingivitis and halitosis were more frequent in the rRT-PCR-positive group than in the rRT-PCR-negative group. Both oral findings could be associated with a lack of oral hygiene, stress, or immunosuppression, which might be some of the most important predisposing factors for the onset of oral lesions in COVID-19 [12]. Gingivitis is a mild periodontal disease characterized by inflammation of the gums and is mainly caused by poor oral hygiene [20]. Periodontal disease is caused by poor oral hygiene, which results in bacteria in dental plaque. The presence of these bacteria causes a local inflammatory reaction and the emergence of neutrophils and other inflammation-promoting cells that receive the mediation of proinflammatory cytokines [21].

Furthermore, evidence suggests that the worsening of SARS-CoV-2 infection in the lung region may be caused by the aspiration of periodontal pathogens, increasing the secretion of inflammatory cytokines such as IL-6 [22]. Our findings are consistent with those reported by Manzalawi et al. [23]., who also observed gingivitis in patients with COVID-19. This association suggests that the debilitating effects of COVID-19 may lead to a neglect of proper oral hygiene measures, contributing to the development of gingivitis. Gingivitis in COVID-19 patients highlights the importance of promoting oral health awareness and emphasizing the significance of maintaining good oral hygiene practices, especially during illness and vulnerability.

In the symptomatic rRT-PCR-negative group, mouth ulcers were self-reported in 12 participants (14.6%) but observed in only three participants (3.7%) during the oral examination. In contrast, only five participants (10.0%) reported mouth ulcers in the rRT-PCR-positive group, with none presenting with such lesions during the clinical oral examination. According to a Spanish study [11], vesicular eruptions (1.6%) were observed in a minority of patients and appeared within the first few days of illness onset. This finding may suggest that vesicular eruptions in the Spanish study and mouth ulcers in our study could be more frequent in the early prodromal phase of the disease.

Glossitis was observed in both groups in our study, with similar relative frequencies. A Spanish study of 666 hospitalized COVID-19 patients presented several oral cavity findings, including transient lingual papillitis (11.5%), aphthous stomatitis (6.9%), and glossitis with lateral indentations (6.6%) [11]. They suggested that these alterations could also be due to specific procedures in hospitalized patients, including wearing ventilation masks. The present study did not observe papillitis; however, isolated glossitis and glossitis with lateral indentations were associated with tongue coating.

Glossitis with lateral indentations (teeth marks on the sides) was described as tongue depapillation with bilateral atrophy of the tongue’s surface in the lateral sides, recently coined “COVID tongue”. Both oral manifestations are related to a particular state of immunosuppression, and stress may play an essential role in the appearance of these oral conditions [12, 24].

Tongue coating (yellow color) was found in four participants (7.4%) in the rRT-PCR-positive group and only two participants (2.4%) in the symptomatic negative group, with no statistically significant difference between the groups. In addition, tongue coating and glossitis with indentations appeared together in some patients and were observed in both groups. According to a retrospective cross-sectional study of tongue features in 1043 patients with COVID-19 [25], with disease progression, the proportion of critically ill patients with yellow tongue coating increased to 62.5%. The authors agreed that fever and infection might cause it to turn yellow. Furthermore, the degree of yellow coating was positively correlated with the degree of lung infection and disease severity. In addition, tongue color change could be attributed to difficulties complying with meticulous oral hygiene measures as the severity of the disease increases and deteriorates [26].

Petechiae were found in the rRT-PCR-positive group (n = 4; 7.4%) and the symptomatic negative group (n = 3; 3.7%). These findings are consistent with previous studies that reported petechiae on the palate, lower lip, and oropharyngeal mucosa during COVID-19 [27, 28]. These authors suggested that oral lesions were associated with COVID-19. In our study, petechiae were found in the soft palate of seven patients. However, the direct effect of SARS-COV-2 on these lesions remains uncertain because conditions other than viral infection, such as adverse drug reactions, may be related to these findings [27].

The findings of oral manifestations in both groups were relatively similar, although it was not possible to detect SARS-CoV-2 using rRT-PCR in all participants. Recent studies suggest that rRT-PCR for SARS-CoV-2 detection should be performed between the third and seventh days of symptom onset to reduce false negative results [29]. Although the participants in our study underwent the rRT-PCR test an average of 4.68 days after the onset of signs and symptoms, the range was from one to 16 days, leading to potential false negative results. Another explanation for false-negative results is the sensitivity (86%) and specificity (96%) of the PCR test used for diagnosis. Therefore, a second sample collection is recommended when the first evaluation is negative to improve the probability of confirming the diagnosis using this molecular technique [30].

Our study has limitations that cannot be generalized to all COVID-19 patients. First, we evaluated only patients with mild-to-moderate disease and not hospitalized patients with more severe forms of COVID-19. Second, we did not analyze the use of medications to treat preexisting diseases, which may have influenced tongue findings and xerostomia. Third, as previously discussed, a second sample was only systematically collected from some symptomatic patients with negative rRT-PCR results.

COVID-19 has a high infectivity rate, mainly because of its spread through respiratory droplets. In addition to studying the role of saliva in the transmission of the virus and as an alternative fluid for diagnosis, one must carefully ensure the identification of saliva quality and the presence of xerostomia. A thorough oral clinical examination can indicate the presence of oral manifestations related to COVID-19 and thus establish immediate measures to reduce the transmission and pathogenicity of SARS-CoV-2.

## Conclusions

In conclusion, a high prevalence of oral manifestations was observed in symptomatic patients with suspected or confirmed COVID-19, highlighting the importance of routine oral examinations by dentists as part of the multidisciplinary care of COVID-19 patients.

## Data Availability

The datasets used and/or analyzed during the current study are available from the corresponding author upon reasonable request.
